# The Oldest Coroner in England

**Published:** 1917-08-18

**Authors:** A. Malcolm

**Affiliations:** 30 Spencer Place, Leeds


### The Oldest Coroner in England.

*To the Editor of* The Hospital.

Sir,—I am ex-home sister of the Middlesex Hospital, London, and continue to take the journal The Hospital. May I correct a paragraph in this week's number, page 372, as to the alleged oldest coroner in England? The late Mr. Taylor was appointed coroner in 1893, and was aged eighty-four. My father, J. C. Malcolm, was appointed coroner for Leeds in September 1876. He is senior Past-President of the Coroners' Society, eighty-six years of age, and still exercising the office of coroner for the city. About twelve years ago he gave up his practice as a solicitor. He is therefore both older in years, and has held the office seventeen more years than the gentleman referred to as the oldest coroner in England. My father has had remarkably good health, seldom needs the aid of his deputy, and since he lost his court clerk, who volunteered at the mobilisation for war, he has not taken any holiday. I am not writing this with any view to publication, but I think you should know the facts.—Yours truly,

A. Malcolm.

30 Spencer Place, Leeds, August 11, 1917.

